# Inhibition of Shh signalling in the chick wing gives insights into digit patterning and evolution

**DOI:** 10.1242/dev.137398

**Published:** 2016-10-01

**Authors:** Joseph Pickering, Matthew Towers

**Affiliations:** Bateson Centre, Department of Biomedical Science, University of Sheffield, Western Bank, Sheffield S10 2TN, UK

**Keywords:** Digits, Limb, Positional information, Self-organization, Shh, Chick development

## Abstract

In an influential model of pattern formation, a gradient of Sonic hedgehog (Shh) signalling in the chick wing bud specifies cells with three antero-posterior positional values, which give rise to three morphologically different digits by a self-organizing mechanism with Turing-like properties. However, as four of the five digits of the mouse limb are morphologically similar in terms of phalangeal pattern, it has been suggested that self-organization alone could be sufficient. Here, we show that inhibition of Shh signalling at a specific stage of chick wing development results in a pattern of four digits, three of which can have the same number of phalanges. These patterning changes are dependent on a posterior extension of the apical ectodermal ridge, and this also allows the additional digit to arise from the Shh-producing cells of the polarizing region – an ability lost in ancestral theropod dinosaurs. Our analyses reveal that, if the specification of antero-posterior positional values is curtailed, self-organization can then produce several digits with the same number of phalanges. We present a model that may give important insights into how the number of digits and phalanges has diverged during the evolution of avian and mammalian limbs.

## Abstract (precis)

**Highlighted Article:** In the chick wing, the relative timing of the specification of antero-posterior positional values and self-organising mechanisms determines digit patterning and identity.

## INTRODUCTION

Various models have been proposed to explain how the digits of the vertebrate limb are specified (Delgado and Torres, 2016). In chick wing and leg buds, a paracrine gradient of Sonic hedgehog (Shh) signalling emanates from the polarizing region and specifies cells with the antero-posterior positional values of three digits – 1, 2 and 3 – in a concentration-dependent manner (Towers et al., 2011, 2012, Fig. 1A,B). In this positional information model, Shh signalling also promotes antero-posterior expansion of the digit-forming field and this generates enough tissue for three positional values to be specified (Towers et al., 2008, Fig. 1A,B). Unlike in the chick wing bud, the polarizing region of the chick leg bud produces a posterior digit, and it is proposed that cells that give rise to this digit are specified with a posterior positional value by the duration of autocrine Shh signalling (Towers et al., 2011, Fig. 1B). An important component of the positional information model is promotion, in which cells are first specified with anterior positional values, before being ‘promoted’ every 4 h to more posterior values (Towers et al., 2011, Fig. 1A,B). This suggests that cells remember their positional values, and then use this information to generate digits with the characteristic number of phalanges at a later stage – presumably via BMP and FGF signalling (Dahn and Fallon, 2000; Sanz-Ezquerro and Tickle, 2003).

**Fig. 1.**
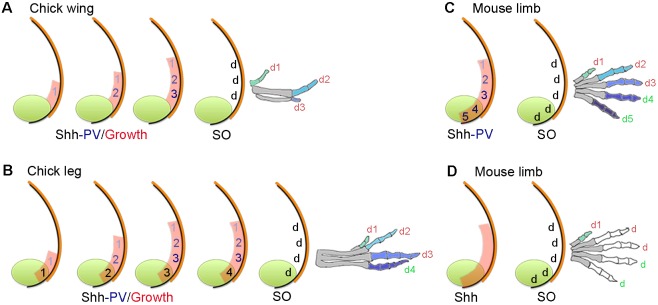
**Positional information and self-organization in digit patterning.** (A) In the chick wing, graded paracrine Shh signalling (numbers shaded blue) from the polarizing region (green) promotes growth of the digit-forming field (red), and in a positional information model, specifies cells with the three positional values (PV) 1, 2 and 3. Cells are specified with anterior positional values and promoted to posterior values every 4 h to give rise to three digits (d) by self-organization (SO). Note that limb buds are not drawn to scale. In all cases, colours on digits indicate a different positional value with which cells were specified, which are interpreted into phalange number (metacarpals are shaded grey). (B) In the chick leg, patterning is as in the chick wing (A) but a digit is derived from the polarizing region (green number), which is specified by the duration of non-graded autocrine Shh signalling (black numbers). (C,D) In the mouse limb, two digits are derived from the polarizing region (note digit 1 positional value is considered independent of Shh), positional values specified by Shh signalling (C) or not specified by Shh signalling (D) and self-organization produces four digits (2-5). Note, in C and D Shh signalling specifies the size of the digit-forming field (red shading).

However, although a model based on positional information can satisfactorily explain how antero-posterior positional values are specified, it fails to explain how digit number is determined. Instead, this is considered to be dependent on an underlying self-organizing mechanism with Turing-type properties (Zwilling, 1964; Wilby and Ede, 1975; Newman and Frisch, 1979), which functions independently of the specification of positional values (Hardy et al., 1995; Elisa Piedra et al., 2000, Fig. 1A,B).

Variations on the positional information model have been proposed for the mouse limb (Fig. 1C) in which Shh signalling is proposed to specify cells with antero-posterior positional values over either a short (biphasic model, Zhu et al., 2008) or a prolonged period (temporal expansion model, Harfe et al., 2004). In the biphasic model, Shh signalling is suggested to have a later role in promoting the survival of digit condensations. In the temporal expansion model, the role of prolonged autocrine Shh signalling is taken into account for specifying polarizing region cells with the positional values of two posterior digits (Fig. 1C). The involvement of a growth phase – in which cells are promoted with antero-posterior positional values – is unclear in both biphasic and temporal expansion models, because digits 2-5 of the mouse limb each have three phalanges and this obscures their identification when digits are lost following the removal of Shh signalling (Scherz et al., 2007; Zhu et al., 2008; Fig. 1C). Note that digit 1, which has two phalanges, is considered to form independently of Shh signalling (Chiang et al., 1996). This raises the possibility that cells that give rise to digits 2-5 of the mouse limb could be specified with a ‘generic’ positional value, perhaps independent of Shh signalling (Delgado and Torres, 2016). Thus, self-organization – which has recently been modelled with a Bmp-Wnt-Sox9 Turing-type network (Sheth et al., 2012; Raspopovic et al., 2014) – would generate four morphologically similar digits, each with three phalanges (Fig. 1D). In this scenario, Shh signalling would only determine the size of the digit-forming field (Fig. 1D) and this fits with the observation that many morphologically similar digits form in *Shh^−/−^/Gli3^−/−^* mouse limbs (Litingtung et al., 2002; te Welscher et al., 2002).

In this study, we show that the inhibition of Shh signalling in the chick wing bud can unexpectedly result in the formation of three digits that have the same number of phalanges – one of which arises from the cells of the polarizing region. We discuss how this process could give insights into how digit and phalange number has diverged in birds and mammals.

## RESULTS

### Shh signalling represses digit formation in the chick wing

**Fig. 2.**
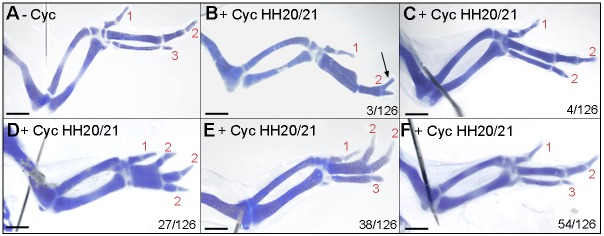
**Effects of cyclopamine on chick wing digit patterning.** Untreated wing (−Cyc) with three digits 1-2-3 (A), wings treated with cyclopamine (+Cyc) at HH20/21 with a 1-2 pattern with a bifurcated digit 2 phalanx (arrow in B; *n*=3/126), with a 1-2-2 pattern (C; *n*=4/126), with a 1-2-2-2 pattern (D; *n*=27/126), with a 1-2-2-3 pattern (E; *n*=38/126) and with normal pattern (F; *n*=54/126). Note, digit 2s bifurcate from a fused metacarpal in D and E. Scale bars: 1 mm.

**Fig. 3.**
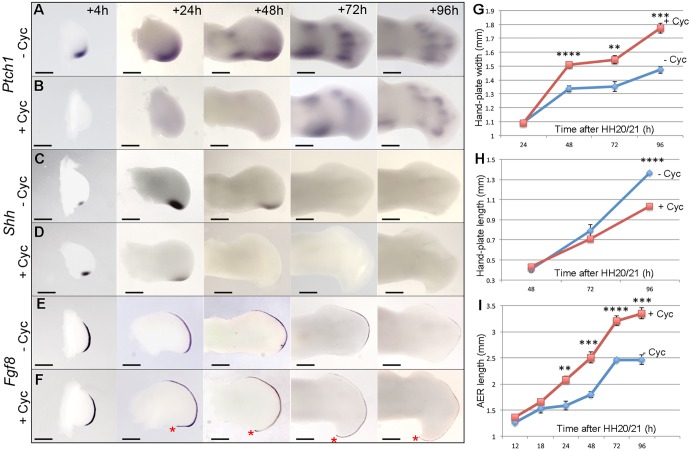
**Effects of cyclopamine on gene expression and the AER.** (A,B) Cyclopamine was applied at HH20/21 and *Ptch1* expression was undetectable after 4 h (*n*=3/3) compared with untreated buds, but detectable at 72 h in cartilage condensations. (C,D) *Shh* expression increased at 4 h but reduced or undetectable at 24 h after cyclopamine treatment (*n*=11/22 reduced; 11/22 undetectable) and after this was undetectable. (E,F) *Fgf8* expression expanded around the posterior margin of cyclopamine-treated buds by 24 h (red asterisks; *n*=7/7). (G) Hand-plate width was significantly greater in cyclopamine-treated wings at 48 h. (H) Hand-plate length was shorter in treated wings at 96 h. (I) The AER was longer in treated wings at 24 h. Error bars indicate s.e. (unpaired *t*-test, **P≤0.005, ***P≤0.0005, ****P≤0.0001). Scale bars: 500 μm (4 h, 24 h), 350 μm (48 h), 200 μm (72 h, 96 h).

The systemic application of cyclopamine (a pharmacological inhibitor of the Shh signalling pathway at the level of Smoothened) to chick embryos results in wings with a reduced number of digits. Application at stage HH18 causes loss of digits 2 and 3, at stage HH20, loss of digit 3 and after stage HH21, all digits were present (Scherz et al., 2007; Towers et al., 2011; Table S1, compare with untreated wings in Fig. 2A). However, application of cyclopamine to chick wing buds can result in bifurcated digits (Fig. 2B; Scherz et al., 2007; Towers et al., 2008) similar to those produced by self-organization (Miura et al., 2006; Sheth et al., 2012). To gain insights into how such bifurcations arise, we carried out an extensive series of experiments in which we applied cyclopamine to embryos between stages HH20 and HH22 (Table S1). Unexpectedly, we observed an unusual range of digit patterns following treatment between stages HH20 and HH21: occasionally wings with a 1-2-2 pattern (Fig. 2C), but frequently wings with four digits in patterns of 1-2-2-2 (Fig. 2D) or 1-2-2-3 (Fig. 2E), in which the digit 2s had distally bifurcated from proximally fused metacarpals. Wings with normal digit patterns were also observed (Fig. 2F), and in 90% of cases, left and right wings had an identical pattern (Table S1). Therefore, the formation of morphologically similar digits following the inhibition of Shh signalling appears to reveal the underlying self-organization mechanism.

### Shh signalling represses formation of the posterior AER

Since it is surprising that the application of cyclopamine to stage HH20/21 chick embryos should result in wings with an additional digit, we examined whether Shh signalling was repressed. By 4 h, expression of *Ptch1*, a direct target of Shh signalling was undetectable (Fig. 3A,B and Table S2), and could only be detected at low levels by 72 h in regions of condensing cartilage – presumably under the regulation of Indian hedgehog (Ihh) signalling (Fig. 3A,B). We also observed that *Shh* expression levels were increased within 4 h, as previously reported (Scherz et al., 2007, Fig. 3C,D). However, only low levels of *Shh* expression could be detected 24 h after cyclopamine treatment and transcripts were undetectable at later stages (Fig. 3C,D and Table S3). This finding indicates that Shh signalling controls its own transcription in an auto-regulatory manner, possibly as part of an intrinsic timing mechanism (Chinnaiya et al., 2014). It was also evident that, by 24 h after cyclopamine treatment, wing buds were broader across the antero-posterior axis, so that by 96 h, the hand-plates of wings were increased in width by 1.2-fold (Fig. 3G), but were 1.32-times shorter in length along the proximo-distal axis (Fig. 3H). In addition, the apical ectodermal ridge (AER) – a thickening of the epithelium that rims the distal tip of the limb bud – was increased in length by 1.4-fold at 96 h (Fig. 3I). However, examination of *Fgf*8 expression showed that the AER was extended posteriorly after 24 h (Fig. 3E,F and Table S4) as was *Fgf4* expression (Fig. S1). In addition, the shape of the cyclopamine-treated wing bud was dramatically altered and formed a pronounced posterior-distal hook. Although the formation of an additional digit is unexpected following cyclopamine application to chick wing buds, the expression of *Gli1*, *Gli2*, *Gli3*, *Grem1* and *Bmp2* recapitulated the patterns reported in previous studies in which Shh signalling was inhibited (Ros et al., 2003; Scherz et al., 2007; Fig. S1). These data show that application of cyclopamine to stage HH20/21 chick embryos rapidly suppresses Shh signalling and also extends the posterior part of the AER around a broadened and shortened bud.

### An additional posterior digit condensation forms after Shh signalling is inhibited

**Fig. 4.**
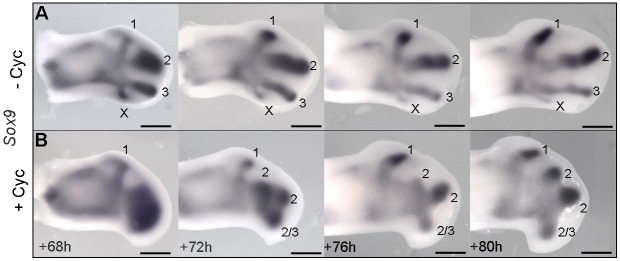
**Shh represses the formation of a posterior digit condensation.** In untreated wings, the posterior-most *Sox9*-expressing digit condensation regresses (indicated by an X in A) but forms either a digit 2 or 3 in wings treated with cyclopamine at HH20/21 (B). Note that resolution of distinct cartilage condensations is delayed in cyclopamine-treated wings. Scale bars: 500 μm.

To determine the origin of the additional digit that forms in the wings of cyclopamine-treated stage HH20/21 chick embryos, we carefully analysed the formation of digit condensations. Surprisingly, *Sox9* expression revealed that, whereas all individual condensations could be clearly observed in untreated wing buds by 68 h (Fig. 4A), only the condensation of digit 1 had segmented from a mass of pre-chondrogenic cells in cyclopamine-treated wing buds (Fig. 4B). Over the next 4 h, this mass of cells resolves into three distinct spots that then segment into three condensations, and although the duplicate digit 2s suggest that an additional central digit condensation gives rise to a digit in cyclopamine-treated wings (Fig. 2D,E), it is, however, an additional posterior condensation, which regresses in normal wing buds (Hinchliffe, 1977; Fig. 4A,B). These data show that inhibition of Shh signalling causes an additional posterior condensation to form in the chick wing.

### The polarizing region forms a digit following inhibition of Shh signalling

**Fig. 5.**
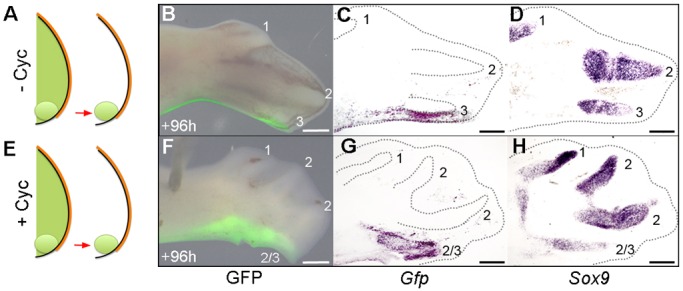
**Shh represses the formation of a digit from the polarizing region.** Grafted HH20 *Gfp*-expressing polarizing regions (A) contribute to the posterior margin of digit 3 in normal wings (*n*=9/9) in whole mounts showing GFP fluorescence (B), and serial sections showing *Gfp* (C) and *Sox9* (D) expression. In HH20/21 cyclopamine-treated wing buds (E), the polarizing region forms a digit (*n*=3/3, F-H). Scale bars: 500 μm.

The fourth digit (running from anterior to posterior) in the mouse limb (Harfe et al., 2004) and chick leg (Towers et al., 2011) arises from the polarizing region. Therefore, we examined if the fourth digit of the pattern in cyclopamine-treated chick wings also arises from the polarizing region. To achieve this, we transplanted polarizing regions from the wing buds of stage HH20 chick embryos that constitutively express green fluorescent protein (GFP; McGrew et al., 2004), in place of the polarizing regions of the wing buds of equivalently staged wild-type embryos, and then applied cyclopamine 2 h later (Fig. 5A,E). As reported previously (Towers et al., 2011), the polarizing region of normal chick wing buds only contributes to soft tissues along the posterior margin of digit 3, as revealed in whole mounts showing GFP fluorescence (Fig. 5B) and consecutive sections hybridized with probes for *Gfp* (Fig. 5C) or S*ox9* (Fig. 5D). However, in cyclopamine-treated embryos (Fig. 5E), GFP-expressing polarizing region cells give rise to the most-posterior digit in wings with patterns of four digits (Fig. 5F-H). This finding shows that inhibition of Shh signalling can cause the polarizing region of the chick wing to give rise to a digit.

### Shh signalling represses polarizing region proliferation and promotes apoptosis

**Fig. 6.**
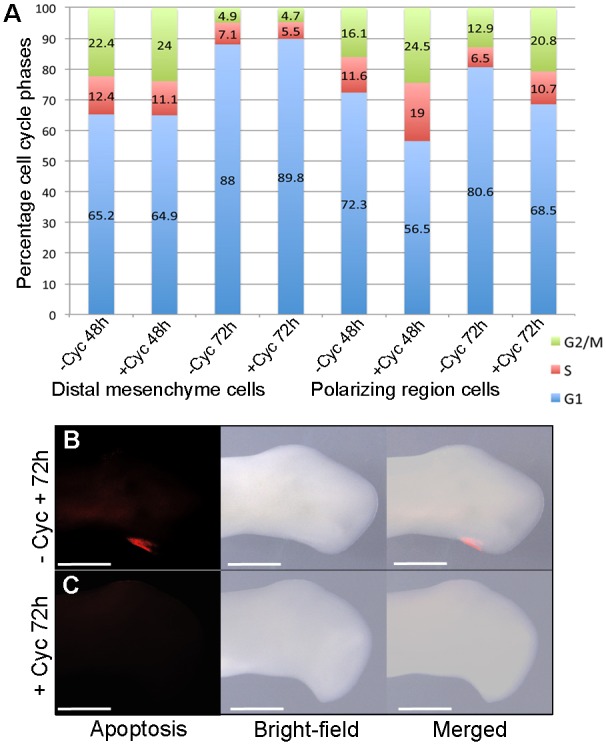
**Shh represses polarizing region proliferation and promotes apoptosis.** Pearson's χ^2^ test reveals no significant difference in G1-phase cells in distal mesenchyme between untreated and HH20/21 cyclopamine-treated embryos at 48 h (*P*=0.732) and 72 h (*P*=0.0842, A). However, there is a significant difference in polarizing region cells at 48 h (*P*<0.0001) and 72 h (*P*<0.0001), indicating an increased rate of proliferation. Apoptosis is detectable in the posterior necrotic zone of untreated wing buds (B, *n*=6/6), but undetectable in HH20/21 cyclopamine-treated wing buds at 72 h (C, *n*=7/7). Scale bars: 500 μm.

To gain insights into the mechanism by which an extra digit forms in the wings of cyclopamine-treated chick embryos, we undertook cell cycle and apoptotic assays. Flow cytometric analyses revealed that there was no significant difference in proliferation parameters of distal mesenchyme cells adjacent to the polarizing region 48 h after cyclopamine application at stage HH20/21 (64.9% vs 65.2% in control embryos) and 72 h (89.8% vs 88.0%; Fig. 6A). G1-phase cells have been shown to deviate by <2% in limb buds of equivalently staged embryos incubated together (Chinnaiya et al., 2014). However, there was a considerable increase in the proliferative potential of polarizing region cells after 48 h (G1 cells, 56.5% vs 72.3% in control embryos) and 72 h (68.5% vs 80.6% ; Fig. 6A). Such proportions of G1-phase cells are normally found in younger wing buds (Chinnaiya et al., 2014), and this reveals that Shh signalling inhibition results in polarizing region cells maintaining an increased proliferative potential for longer. In addition, although the posterior necrotic zone that normally overlaps the proximal part of the polarizing region could be detected in untreated wing buds (Fig. 6B), this region of apoptosis was undetectable by 72 h after cyclopamine treatment at stage HH20/21 (Fig. 6C). Therefore, these data indicate that inhibition of Shh signalling increases proliferation relative to apoptosis in the posterior part of the chick wing bud, and this is associated with the polarizing region producing a digit.

### The AER supports polarizing region digit development

**Fig. 7.**
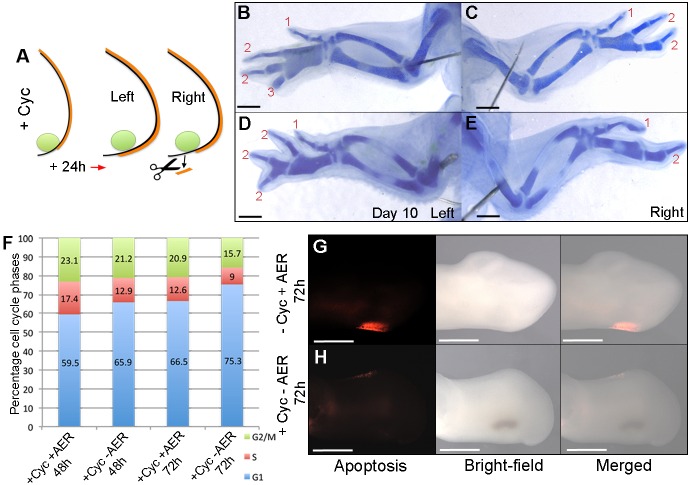
**AER supports polarizing region digit formation.** (A) Cyclopamine was applied at HH20/21 and posteriorly extended AER removed in right-hand wing buds after 24 h. 1-2-2 (C) or 1-2 (E) digit patterns develop. Note four digits in left wings of same embryos (B,D; *n*=15/15; see Table S5). Pearson's χ^2^ test reveals a significant increase in G1-phase cells in the polarizing region at 48 h (*P*<0.0001) and 72 h (*P*<0.0001) in HH20/21 cyclopamine-treated wing buds with the posterior AER removed compared with HH20/21 cyclopamine-treated wing buds with an intact posterior AER (F), indicating a reduced rate of proliferation. Apoptosis is detectable in the posterior necrotic zone of untreated wing buds (G, *n*=6/6) but undetectable in HH20/21 cyclopamine-treated wing buds in which the AER was removed (H, *n*=8/8). Scale bars: 1 mm (B-E), 500 μm (G,H).

Since the AER supports the development of posterior digits that arise from the polarizing region of the mouse limb (Zúñiga et al., 1999; Harfe et al., 2004), we examined if this is also the case in cyclopamine-treated chick wings. To address this, we applied cyclopamine to stage HH20/21 embryos and then removed the extended region of AER in right-hand wing buds after 24 h (Fig. 7A). Analyses of day 10 skeletons showed that this manipulation resulted in the loss of the most-posterior digit to produce wings with a 1-2-2 pattern (Fig. 7C); note 1-2-2-3 pattern in left control wing (Fig. 7B and Table S5). In addition, the adjacent digit also frequently failed to form and this resulted in wings with a 1-2 pattern, thus suggesting that the removal of the AER also affects the formation of digits that would normally have bifurcated from fused metacarpals (Fig. 7E); note bifurcated digits in 1-2-2-2 pattern in left control wing (Fig. 7D and Table S5). Moreover, removal of the extended region of the posterior AER in the right-hand wing buds of stage HH20/21 cyclopamine-treated embryos resulted in significantly more G1-phase polarizing region cells after 48 h (65.9%) and 72 h (75.3%) than in left wing buds in which the AER was left intact (59.5% and 66.5%, respectively; Fig. 7F). However, although G1-phase values of polarizing region cells in cyclopamine-treated wing buds with the posterior AER removed returned to close to those of the polarizing regions of untreated wings buds (72.3% and 80.6%; Fig. 6A), the removal of the AER did not result in a recovery of apoptosis in the posterior necrotic zone compared with untreated wing buds (Fig. 7G,H). Taken together, these data indicate that the AER supports the formation of an extra digit from the polarizing region of cyclopamine-treated wing buds, although additional factors downstream of Shh signalling are likely to impair the behaviour of posterior cells.

### AER length is proportional to polarizing region digit number

**Fig. 8.**
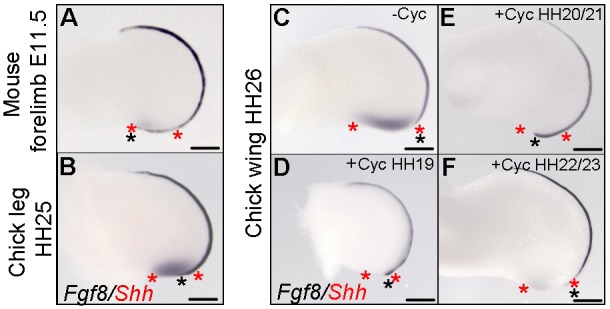
**AER length is proportional to polarizing region digit number.** (A) Weak *Shh* expression in the E11.5 mouse fore-limb bud polarizing region (red asterisks) completely overlaid by AER as observed by *Fgf8* expression (black asterisk indicates limit of the AER, *n*=6/6). (B) *Shh* expression in the HH25 chick leg bud polarizing region partially overlaid by the *Fgf8*-expressing AER (*n*=8/8). (C) *Shh* expression in the HH26 chick wing bud polarizing region not overlaid by the *Fgf8*-expressing AER (*n*=8/8). (D) *Shh* expression is undetectable in the HH26 wing bud polarizing region treated with cyclopamine at HH19 slightly overlaid by the *Fgf8*-expressing AER (*n*=4/4). (E) *Shh* expression is undetectable in the HH26 wing bud polarizing region treated with cyclopamine at HH20/21 partially overlaid by the *Fgf8*-expressing AER (*n*=13/13). (F) *Shh* expression is weak in the HH26 chick wing bud polarizing region treated with cyclopamine at HH22/23 not overlaid by the *Fgf8*-expressing AER (*n*=5/5). Note the position of polarizing region cells that would have expressed *Shh* in D and E estimated by the shape of wing buds compared with normal wing buds. Scale bars: 350 μm.

Since the AER supports the development of a digit in cyclopamine-treated chick wings, we examined if a correlation exists between the length of the posterior AER and the number of digits that the polarizing region produces. *Shh* and *Fgf8* double *in situ* hybridization demonstrated that the AER completely overlies the polarizing region of the E11.5 mouse fore-limb bud that gives rise to two digits (Fig. 8A), but partially overlies the polarizing regions of both the stage HH25 chick leg (Fig. 8B) and the stage HH26 chick wing (treated with cyclopamine at stage HH20/21) that give rise to one digit (Fig. 8E). However, the AER fails to reach the polarizing region of the untreated stage HH26 chick wing bud (Fig. 8C). It should be noted that treatment with cyclopamine at earlier (HH19) or later stages (HH22/23) failed to extend the AER over the polarizing region (Fig. 8D,F), indicating that this could be why treatment at only stage HH20/21 results in the formation of an extra posterior digit. Therefore, the number of digits produced by the polarizing region is proportional to the posterior limit of the AER.

## DISCUSSION

**Fig. 9.**
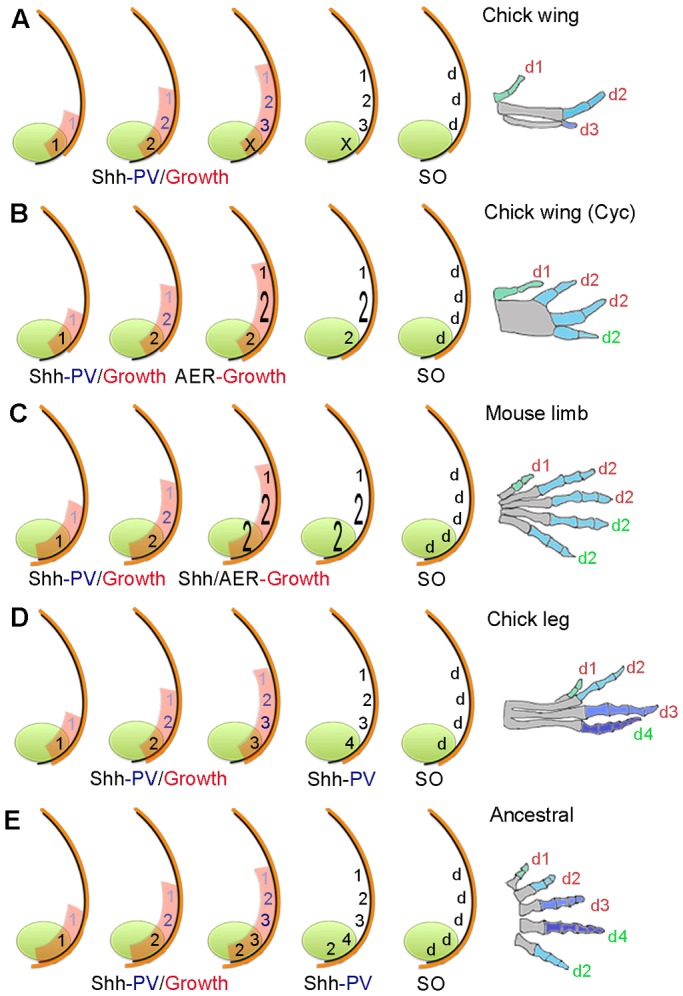
**Timing of positional information and self-organization in digit patterning.** (A) In the chick wing, graded paracrine Shh signalling (numbers shaded blue) from the polarizing region (green) promotes antero-posterior expansion (red) and specifies cells with three positional values (PV) 1, 2 and 3. Cells specified with anterior positional values are promoted to more-posterior values every 4 h and give rise to three digits (d) by self-organization (SO). In parallel, non-graded autocrine Shh signalling (black numbers) specifies polarizing region cells with a positional value, which then fail to form a digit (X). In all cases, colours on digits indicate a different positional value with which cells were specified, which are interpreted into phalange number (metacarpals are shaded grey and limb buds not drawn to scale). (B) Chick wing treated with cyclopamine (Cyc) at HH20/21. Specification is stopped when cells have the positional value of a digit 1 and 2, then posteriorly extended AER (orange) promotes antero-posterior expansion to enlarge the field of cells specified with the positional value of a digit 2 (enlarged 2) to give rise to two digits by self-organization, note fused metacarpals. In addition, polarizing region cells specified with a digit 2 positional value give rise to a digit (green number). Note, 1-2-2-3 pattern (Fig. 2E) could be explained by asynchronous promotion by paracrine and autocrine Shh signalling (Towers et al., 2011). (C) In the mouse limb, patterning is as in the cyclopamine-treated chick wing (B), but the AER extends further and allows polarizing region cells specified with a digit 2 positional value (enlarged 2) to form two digits. Shh has a later role in promoting antero-posterior expansion to allow digits 2-5 to form. (D) In the chick leg, patterning is as in the wing (A), but autocrine Shh signalling continues to promote polarizing region cells with more-posterior positional values, which then produce a digit (Towers et al., 2011). (E) In the ancestral limb, patterning is as in chick leg (D), but most-posterior polarizing region cells specified with a digit 2 positional value could become refractory to Shh signalling at an early stage, as in the mouse limb (Ahn and Joyner, 2004).

We have revealed that inhibition of Shh signalling in the chick wing bud extends the AER posteriorly and this results in a pattern of four digits, three of which can have the same number of phalanges (Fig. 9A,B). We predict that the precise point at which autocrine Shh signalling is inhibited can result in polarizing region cells giving rise to either a digit 2 or a digit 3 (Fig. 9B, Fig. 2D,E). In addition, we showed that in normal development, polarizing region digit formation is prevented because a rapid rate of proliferation is not maintained in the absence of an overlying AER (Fig. 9A). However, an intrinsic cell cycle timing mechanism controlled by Shh signalling in polarizing region cells could also contribute to reduced proliferation (Chinnaiya et al., 2014). Furthermore, we showed that Shh signalling is required for maintenance of apoptosis in the posterior necrotic zone of the chick wing, which could contribute to digit loss (Sanz-Ezquerro and Tickle, 2000). Thus, removal of the posteriorly extended AER following Shh inhibition failed to restore the posterior necrotic zone and our results are therefore consistent with the finding that Shh signalling directly induces apoptosis in the mesenchyme (Sanz-Ezquerro and Tickle, 2000), which is likely to occur via Bmp2 signalling (Bastida et al., 2009).

During normal chick wing development, Shh signalling directly promotes antero-posterior expansion, and this produces enough cells for the antero-posterior positional values of three digits to be specified (Towers et al., 2008; Fig. 9A). However, we revealed that if Shh signalling is inhibited during the time that positional values are specified, a switch to AER-dependent antero-posterior expansion occurs (Fig. 9B). This allows cells adjacent to the polarizing region, which are specified with a digit 2 positional value, to give rise to two digits that bifurcate from a fused metacarpal by self-organization (enlarged 2 in Fig. 9B). In this paper, we have only considered the interpretation of antero-posterior positional values into phalange number and not other morphological differences that give the digits their distinct identities.

We also observed that Shh inhibition reduces growth along the proximo-distal axis, and this occurs without changes in proliferation in the distal mesenchyme adjacent to the polarizing region. Similarly, in *talpid^3^* mutant chick wing buds, in which the defective response to Shh signalling also reveals the underlying self-organizing system as evidenced by multiple fused digits, growth is also reduced along the proximo-distal axis of broadened buds – and this occurs in concert with an extension of the AER (Ede and Kelly, 1964; Davey et al., 2006). Taken together, we propose that the bifurcated skeletal elements present in cyclopamine-treated chick wings and also in *talpid^3^* chick wings are caused by FGF signalling from the AER influencing directional growth (Ede and Flint, 1975). Indeed, dye-labelled cells proliferate towards a bead soaked in FGF4 implanted into the chick wing bud (Li and Muneoka, 1999). In addition, our observation that Shh signalling limits the posterior extent of the AER is consistent with the finding that a polarizing region graft made distally to a chick wing bud causes the overlying AER to regress (Saunders and Gasseling, 1968), although it maintains the adjacent AER through a well-characterized reciprocal feedback loop (Laufer et al., 1994; Niswander et al., 1994; Zúñiga et al., 1999).

### Implications for mouse limb digit patterning

In view of our chick wing data, we suggest that Shh-dependent promotion of antero-posterior positional values could stop in the mouse limb bud when cells are specified with a digit 2 value (Fig. 9C). Further antero-posterior expansion could then allow four digits, each with the same number of phalanges, to form by self-organization (Fig. 9C). In addition, the AER of the mouse limb extends around the polarizing region and this could allow it to form two digits (Harfe et al., 2004). In a similar manner to the chick wing, the posterior limit of the AER of the mouse limb is determined by Shh signalling, as revealed when Smoothened function is specifically removed in this structure (Bouldin et al., 2010).

Our model can be contrasted with a previous study on the mouse limb, in which it was suggested that Shh signalling rapidly specifies cells with the positional values of all five digits, and then Shh-dependent growth permits digit condensations to form in the order that they develop (Zhu et al., 2008). This interpretation was reached because the early removal of Shh signalling appeared to allow posterior digits to form in the absence of more-anterior digits. Instead, we suggest that only anterior positional values had been specified and that digits failed to develop because reduced Shh-dependent antero-posterior expansion depleted the number of cells available to undergo self-organization.

A prediction of our model for the mouse limb is that cells become refractory to levels of Shh signalling required for further promotion of antero-posterior positional values, but not for antero-posterior expansion. Indeed, it has been demonstrated that cells in the mouse limb bud respond to Shh signalling in a linear, rather than a graded manner, and this is consistent with the specification of a single antero-posterior positional value (Ahn and Joyner, 2004).

### Implications for digit evolution in birds and mammals

The fore-limbs of the common ancestor of birds and mammals had five digits with a characteristic number of phalanges (Hopson, 1995; 2-3-4-5-3 running from anterior-posterior; see Fig. S2 for extended discussion, note hind-limb pattern is 2-3-4-5-4). Interestingly, the four digits of the chick leg have maintained the ancestral phalange number and could therefore give insights into the ancestral patterning mechanism (Fig. 9D,E). Thus, we previously showed that a parallel process of paracrine and autocrine Shh signalling specifies cells with the four antero-posterior positional values of the chick leg digits (Towers et al., 2011; Fig. 9D) and therefore potentially the ancestral limb (Fig. 9E). Moreover, we speculate that the fifth digit of the ancestral limb was derived from cells that rapidly became refractory to Shh signalling, as in the mouse limb (Ahn and Joyner, 2004), but at a point at which they were specified with the positional value of a digit 2 in the fore-limb (Fig. 9E), or a digit 3 in the hind-limb.

During the evolution of the bird wing, two posterior digits (4 and 5) were lost, and then the number of phalanges in digit 3 gradually reduced (Sereno, 1999; Padian, 2004; Fig. S2 and Fig. 9A). Our data suggest that the specification of the positional values of digits 1, 2 and 3 has been conserved throughout the evolution of the bird wing (Fig. S2 and Fig. 9A,E). By contrast, during the evolution of the general mammalian digit pattern, the number of phalanges was reduced to three in digits 3 and 4, thus making digits 2-5 morphologically similar (Hopson, 1995; Fig. S2 and Fig. 9C). Therefore, we speculate that this pattern arose as a consequence of the truncated specification of antero-posterior positional values, followed by the self-organization of cells into four digits, each with the same number of phalanges (Fig. 9C).

## MATERIALS AND METHODS

### Chick husbandry

GFP-expressing (Roslin Institute, Edinburgh, UK) and wild-type fertilized Bovans brown chicken eggs (Henry Stewart; MedEggs, Heath Farm House, Norfolk, UK) were incubated, opened and staged according to Hamburger and Hamilton (1951). All experiments involving live chick embryos conformed to the relevant regulatory standards (University of Sheffield).

### Mouse and chick embryo dissections

Embryos were dissected from their membranes in DMEM (Gibco) and were fixed overnight in 4% paraformaldehyde (PFA) at 4°C.

### Polarizing region grafts

Embryos were dissected in DMEM (Gibco) and wing polarizing regions removed using fine tungsten needles, grafted to the appropriate location of stage-matched host limb buds and held in place with 25 μm platinum pins. Polarizing region tissue was removed in reference to patterns of *Shh* expression.

### Apical ectodermal ridge removal

The posterior apical ectodermal ridge was visualized by staining with 1.5% Nile Blue solution (Sigma). Then the extended posterior region of apical ectodermal ridge was teased away from the mesenchyme using sharpened tungsten needles and then cut using micro-dissection scissors.

### Shh signalling inhibition

Cyclopamine (Sigma) was suspended in control carrier [45% 2-hydropropyl-β-cyclodextrin in PBS (Sigma)] to a concentration of 1 mg/ml and 4 μl was pipetted directly onto embryos over the limb bud, after removal of vitelline membranes. Note that in all cases, untreated wings were treated with 2-hydropropyl-β-cyclodextrin only.

### Alcian Blue skeletal preparations

Embryos were fixed in 90% ethanol for 2 days then transferred to 0.1% Alcian Blue (Sigma) in 80% ethanol/20% acetic acid for 1 day, before being cleared in 1% KOH.

### Whole mount *in situ* hybridization

Embryos were fixed in 4% PFA overnight at 4°C, dehydrated in methanol overnight at −20°C, rehydrated through a methanol/PBS series, washed in PBS, then treated with proteinase K (Sigma) for 20 min (10 μg/ml), washed in PBS, fixed for 30 min in 4% PFA at room temperature and then prehybridized at 69°C for 2 h (50% formamide/50% 2× SSC). Antisense DIG-labelled mRNA probes (1 μg) was added in 1 ml of hybridization buffer (50% formamide/50% 2× SSC) at 69°C overnight. Embryos were washed twice in hybridization buffer, twice in 50:50 hybridization buffer and MAB buffer, and then twice in MAB buffer, before being transferred to blocking buffer [2% blocking reagent (Roche), 20% lamb serum (Sigma) in MAB buffer] for 2 h at room temperature. Embryos were transferred to blocking buffer containing anti-digoxigenin antibody (1:2000, Roche, 11093274910) at 4°C overnight, then washed in MAB buffer overnight before being transferred to NTM buffer (100 mM NaCl, 100 mM Tris-HCl, pH 9.5, 50 mM MgCl_2_) containing Nitro Blue tetrazolium/BCIP and mRNA distribution was visualized using a Leica MZ16F microscope. Antisense probes for chick genes used in this study: *Shh* (plasmid-pSport, restriction enzyme-Sal1, RNA polymerase-Sp6), *Fgf8* (pBS, *Not*1, T7), *Ptch1* (pBS, *Sal*1, T3), *Sox9* (pGEM, *Nco*1, Sp6), *Gli1* (pBS, *Kpn*1, T3), *Gli2* (pBS, *Nco*1, T7), *Gli3* (pBS, *Xba*1, T3), *Fgf4* (pBS, *Bam*H1, T7), *Grem1* (pGEM, *Sal*1, T7), *Bmp2* (pBS, *Hin*dIII, T3), *Gfp* (pBS, *Bam*H1, T7). Antisense probes for mouse genes used in this study: *Fgf8* (pBS, *Hin*dIII, T3), *Shh* (pBS, *Hin*dIII, T3); note that all plasmids were obtained from the Cheryll Tickle lab.

### Section *in situ* hybridization

Wing buds were removed from chick embryos and fixed overnight in 4% PFA at 4°C then washed in PBS and left in 30% sucrose solution overnight at 4°C and then mounted directly onto a chuck in OCT (VWR) and sectioned (14 μm) on a cryostat (Bright). Sections collected on superfrost slides (VWR) were dried out and frozen overnight at −20°C, then fixed for 10 min, washed in PBS, treated with an acetylating mix (triethanolamine/acetic anhydride) for 10 min, washed in PBS and placed in a Coplin jar with prehybridization solution (50% formamide, 5× SSC) for 2 h at 69°C. Sections were hybridized with probes for *Gfp* and *Sox9* overnight at 69°C, washed for 1 h at 69°C in 50% formamide/5× SSC, then in 50% formamide/2× SSC and finally, two washes in TBS. Blocking buffer (TBS and 10% goat serum) was applied to sections for 40 min, then replaced with blocking buffer containing anti-digoxigenin antibody (1:2000, Roche, 11093274910) for 80 min. Sections were washed in TBS and then in NTM buffer, and then with NTM buffer with NBT/BCIP. mRNA distribution was visualized using an Olympus BX60 microscope.

### Apoptosis analyses

Chick wing buds were dissected in PBS and transferred to Lysotracker (Life Technologies, L-7528) PBS solution (1:1000) in the dark. Wing buds were incubated for 1 h at 37°C, washed in PBS, and fixed overnight in 4% PFA at 4°C. Wing buds were then washed in PBS and progressively dehydrated in a methanol series.

### Flow cytometry

Distal mesenchyme or polarizing region tissue from replicate experiments (14 wing bud samples from 7 embryos) was dissected into 100 μm blocks in ice cold PBS under a Leica MZ16F microscope using a fine surgical knife and pooled. Blocks were digested into single cell suspensions with 0.5% trypsin (Gibco) for 30 min at room temperature. Cells were briefly washed twice in PBS, fixed in 70% ethanol overnight, washed in PBS and resuspended in PBS containing 0.1% Triton X-100, 50 µg ml^−1^ propidium iodide and 50 µg ml^−1^ RNase A (Sigma). Dissociated cells were left at room temperature for 20 min, cell aggregates were removed by filtration and single cells analysed for DNA content with a FACSCalibur flow cytometer and FlowJo software (Tree Star). Based on ploidy values, cells were assigned to G1-, S- or G2/M-phase and this was expressed as a percentage of the total cell number (approximately 5000 in each case). Statistical significance of numbers of cells between pools of dissected wing bud tissue (14 in each pool) was determined by Pearson's χ^2^ tests to obtain two-tailed *P*-values [significantly different being a *P*-value of less than 0.05 (Chinnaiya et al., 2014) for statistical comparisons of cell cycle parameters between the wing buds of embryos incubated together].
